# Cardiac metastatic melanoma presenting with ventricular tachycardia: a multimodality imaging evaluation case report

**DOI:** 10.1093/ehjcr/ytae505

**Published:** 2024-09-14

**Authors:** Hussain Mirza Khalid, Yomary Jimenez, Wei Wang, Bruno Hochhegger, Mohammad Al-Ani

**Affiliations:** Department of Internal Medicine, Division of Cardiovascular Medicine, University of Florida Health, 1600 SW Archer Rd, Gainesville, FL 32608, USA; Department of Internal Medicine, Division of Cardiovascular Medicine, University of Florida Health, 1600 SW Archer Rd, Gainesville, FL 32608, USA; Department of Internal Medicine, Division of Cardiovascular Medicine, University of Florida Health, 1600 SW Archer Rd, Gainesville, FL 32608, USA; Department of Radiology, University of Florida Health, 1600 SW Archer Rd, Gainesville, FL 32608, USA; Department of Internal Medicine, Division of Cardiovascular Medicine, University of Florida Health, 1600 SW Archer Rd, Gainesville, FL 32608, USA

**Keywords:** Melanoma, Cardiac mass, Imaging, Case report

## Abstract

**Background:**

Cardiac tumours are rare; secondary, metastatic cardiac tumours are 22–132 times more common than primary cardiac tumours. Multimodality imaging can elucidate the mass anatomy, composition, haemodynamic consequences, and guide management plan.

**Case summary:**

We present a case of large left ventricular mass presenting with unstable ventricular tachycardia. We describe the cardiac magnetic resonance imaging, transthoracic echocardiography, and computed tomography findings used to assist in characterizing the left ventricular mass. We describe the multidisciplinary discussion involved in diagnosis, surgical biopsy, and treatment, and follow-up of cardiac metastatic melanoma.

**Discussion:**

Metastatic melanoma should be within the differential for cardiac masses. Any patient presenting with a cardiac mass should be asked about history of skin malignancy. Multimodality imaging is crucial to diagnosis, staging, haemodynamic assessment, interventional and surgical planning, and assessment of response to therapy.

**Learning points**

- Metastatic melanoma commonly involves the heart. Although more commonly affecting the right heart, cardiac melanoma can present in the left ventricle and involve any cardiac structure.
- Multimodality cardiac imaging provides complimentary information—allowing for tissue characterization (particularly with cardiac MRI), assessment of effects on valvular function and ventricular outflow tract obstruction (particularly with echocardiography), and assessment of surrounding extracardiac structures (particularly with computed tomography).
- Histologic characterization of suspected malignant cardiac masses can be accomplished least invasively if there are sites of extracardiac involvement amenable to sampling, or if there is associated pericardial fluid that can be sampled by pericardiocentesis. Surgical excision of cardiac masses may be indicated for masses with solitary or localized involvement, significant haemodynamic compromise, or potential for embolization.

## Introduction

Cardiac tumours are rare. Secondary metastatic cardiac tumours are more common than primary cardiac tumours.^1^ Although lung, breast, and oesophageal cancers represent the most common source of metastatic cardiac tumours due to overall higher prevalence of these cancers, metastatic melanoma has a high propensity for cardiac involvement.^1^ Multimodality imaging is essential in the evaluation of cardiac tumours, and we present a case that outlines its crucial role in the evaluation and management of a patient presenting with arrhythmia and an incidental cardiac tumour.

## Summary figure

**Table:** 

2015	Excision of right shoulder melanoma
September 2022—admission to outside hospital	Presentation to outside hospital with chest pain, palpitations, monomorphic ventricular tachycardia, elevated high-sensitivity troponin
	Coronary angiography demonstrating normal coronary arteries
	CMR demonstrating left ventricular mass
	Hospital transfer for further evaluation
September 2022	TTE w/ultrasound contrast demonstrating mild mitral regurgitation and mild contrast uptake in a non-mobile LV septal mass
	CT C/A/P with contrast without evidence of a separate primary malignancy
	Multidisciplinary discussion regarding diagnosis of the LV mass and treatment strategies
	VT storm refractory to antiarrhythmic therapy requiring stellate ganglion block
	Surgical biopsy and septal myomectomy 2 cm × 1 cm via open sternotomy; pathology specimens consistent with metastatic melanoma
	Dual-chamber ICD placement
	Ipilimumab and nivolumab treatment imitated for planned 3 cycles
	Discharge from hospital
October 2022	Whole body 18-FDG-PET with no other sites of avidity
December 2022	Repeat CMR demonstrating stable size of the cardiac mass
January 2023	Switch to monthly nivolumab therapy ICD discharge ×4 for VT Radiofrequency ablation of VT originating from three different foci in the interventricular septum and right ventricular apex Repeat CMR demonstrating stable size of the mass
April 2023	PET/CT demonstrating decreased size of the LV mass Continued monthly nivolumab therapy
August 2023	Repeat PET/CT and CMR demonstrating increased size of the cardiac mass
September 2023	Initiation of Opdualag (nivolumab/relatlimab)

## Case presentation

**Figure 1:**
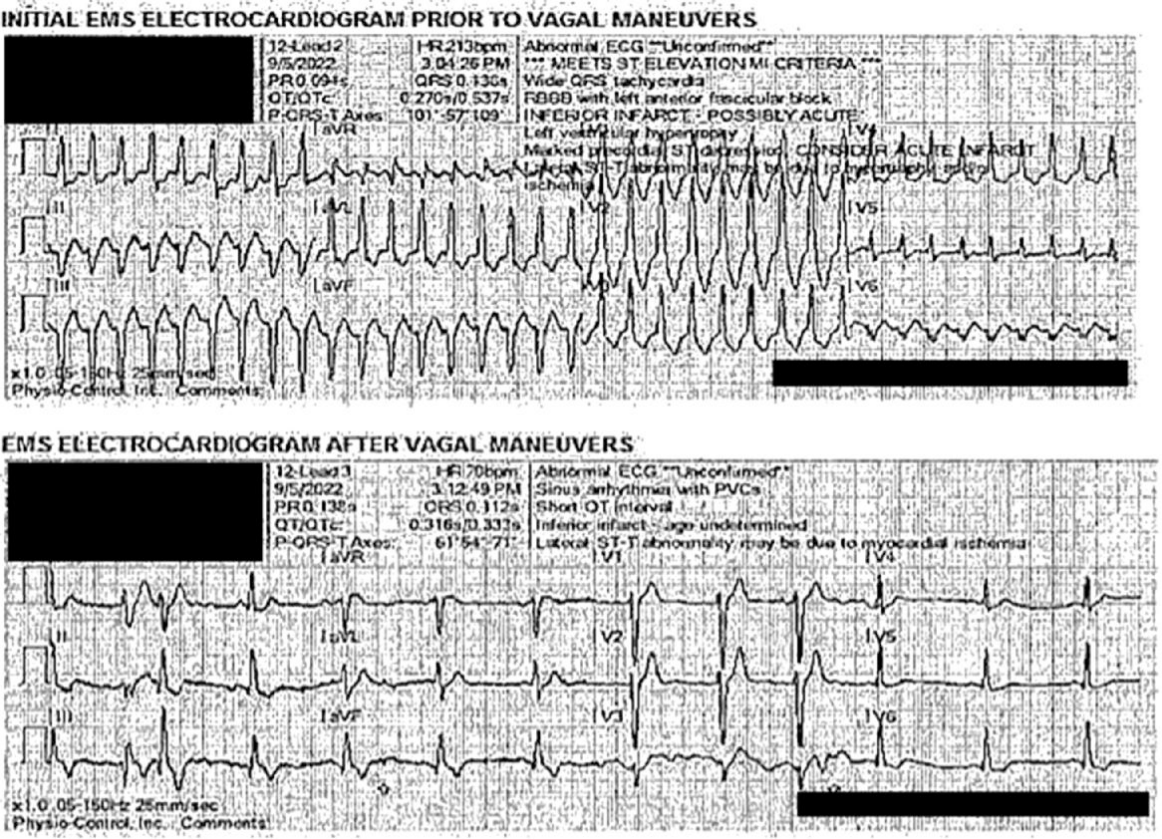
Emergency medical services ECG demonstrating monomorphic ventricular tachycardia at 213 b.p.m. (cycle length 282 ms). Right bundle branch block morphology of the tachycardia not present while the patient was in normal sinus rhythm, monophasic R-wave in lead V1, and R:S ratio of <1 in lead V6 suggests VT as opposed to SVT with aberrancy. The origin of the VT was suspected to be the mid-to-basal left ventricular posterior wall based upon the RBBB morphology, superior QRS axis, and R-wave dominance in the precordial leads V3–V5.

A 51-year-old female with history of right shoulder melanoma excision seven years prior presented with chest pain and palpitations. Electrocardiogram (ECG) obtained by emergency medical services demonstrated a wide-complex tachycardia that reportedly resolved after vagal manoeuvres (*Figure 1*). The patient subsequently had normal vital signs and physical exam upon admission. Laboratory studies demonstrated normal blood counts and metabolic panels. High-sensitivity troponin I peaked at 935 ng/L (normal < 49 ng/L). Chest X-ray did not reveal any acute cardiopulmonary process. The cardiology team determined the arrhythmia was monomorphic ventricular tachycardia (VT) due to the right bundle branch block morphology of the tachycardia not present while the patient was in normal sinus rhythm, monophasic R-wave in lead V1, and R:S ratio of <1 in lead V6. Invasive coronary angiography demonstrated normal coronary arteries. Cardiac magnetic resonance (CMR) imaging was performed to evaluate for myocardial scarring or infiltration, and revealed a left ventricular (LV) mass, prompting the patient’s transfer to our institution.

**Figure 2:**
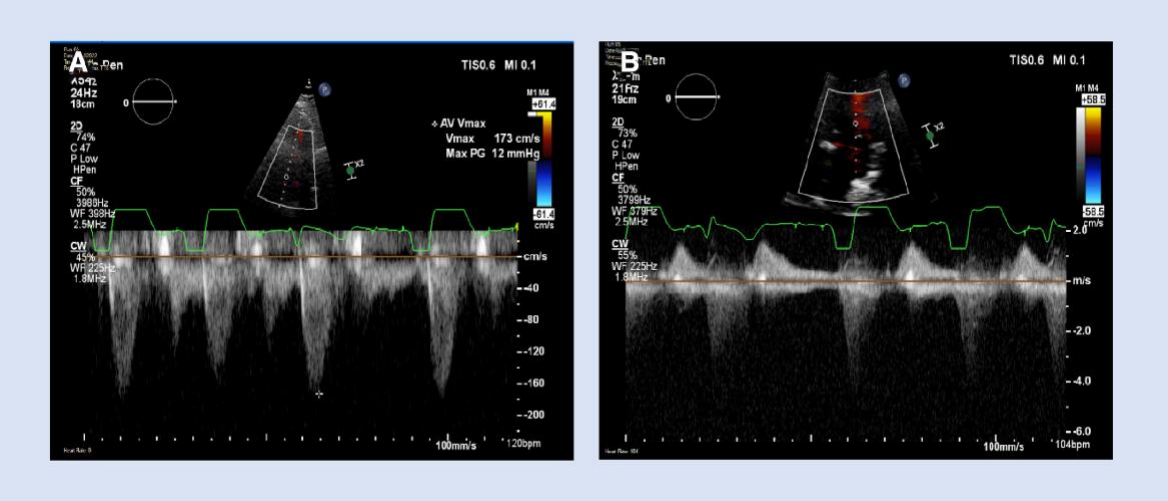
(*A*) Continuous-wave Doppler (CWD) across the aortic valve in the A5C view demonstrating no haemodynamically significant obstruction by the cardiac mass. (*B*) CWD in the A2C view demonstrating mild mitral regurgitation jet.

Transthoracic echocardiogram (TTE) demonstrated small LV chamber size without evidence of LV outflow tract or intracavitary obstruction by the cardiac mass (*Figure 2A*, Supplementary material online, *Video S1*). Colour and Spectral Doppler demonstrated mild mitral regurgitation (*Figure 2B*, Supplementary material online, *Video S2*). Intravenous Definity® contrast was administered for LV chamber opacification and revealed normal regional wall motion, normal biventricular systolic function, and a LV cardiac mass involving the mid-to-basal septum and inferior walls with mild contrast enhancement (see Supplementary material online, *Videos S1* and *S3*–*S5*). Given the LV septal location, normal regional wall motion, no independent mobility of the mass, and evidence of contrast uptake, it was unlikely that this cardiac mass represented thrombus, cyst, myxoma, fibroelastoma, or vegetation.

**Figure 3:**
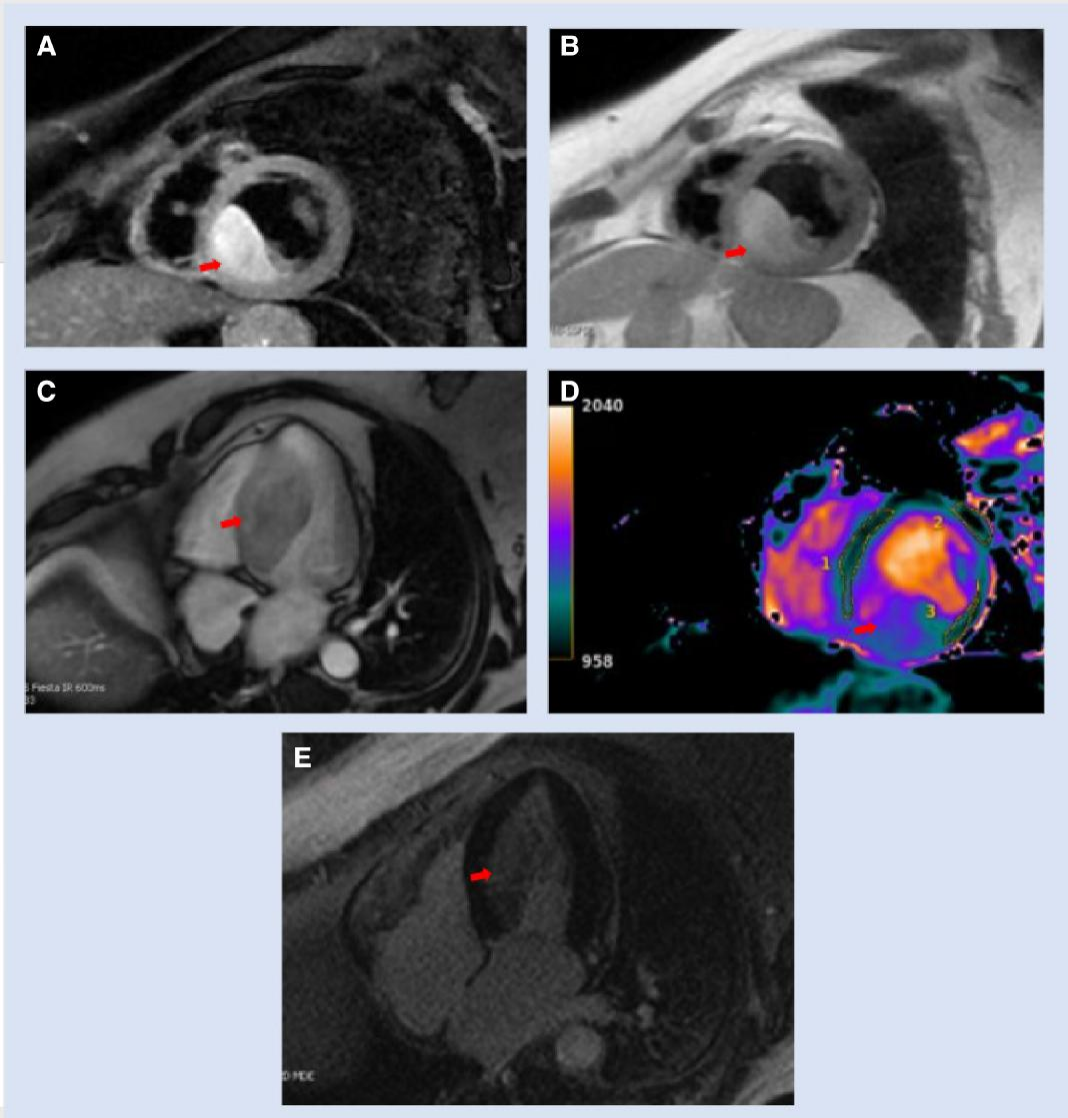
Arrows in *A–E* point to cardiac mass. (*A*) Short-axis T2W BB SPIR demonstrating hyperintense signal in the cardiac mass. TE 51.4, TR 2045. (*B*) Short-axis T1W BB SSFE demonstrating hyperintense signal in the cardiac mass. TE 36.2, TR 2010.2. (*C*) Four-chamber FIESTA with IR of 600 ms demonstrating that the cardiac mass had isointense signal relative to the myocardium. TE 1.8, TR 4.1. (*D*) Short-axis T1 map demonstrated elevated T1 relaxation time in the cardiac mass relative to myocardium (representative myocardium outlined and numbered ‘1’, ‘2’, and ‘3’). (*E*) Four-chamber MDE sequence demonstrating diffuse MDE in the cardiac mass. TE 1.54, TR 5.688.

Outside hospital CMR (GE Optima 450 W 1.5 T) demonstrated normal biventricular systolic function and regional wall motion (see Supplementary material online, *Video S6*). A 5.9 cm × 4.4 cm × 2.6 cm LV mass involving the mid-to-basal inferoseptal and inferior walls was visualized (see Supplementary material online, *Videos S6*–*S8*). Eccentric mitral regurgitation was noted due to restricted motion of the posterior leaflet (see Supplementary material online, *Video S7*). T2-weighted (T2W) black blood (BB) fat-suppressed Spectral Presaturation with Inversion Recovery (SPIR) sequences demonstrated hyperintense signal in the cardiac mass compared to myocardium suggesting the mass was not a lipoma (*Figure 3A*). T1-weighted (T1W) BB Single Shot Fast Spin Echo (SSFSE) demonstrated hyperintense signal in the cardiac mass relative to the myocardium (*Figure 3B*). Fast Imaging Employing Steady-state Acquisition (FIESTA) imaging with inversion recovery (IR) of 600 ms demonstrated that the cardiac mass had isointense signal relative to the myocardium suggesting that this mass was not thrombus (*Figure 3C*). T1 map demonstrated elevated T1 relaxation time in the cardiac mass relative to myocardium (*Figure 3D*). FGRE Time Course sequences demonstrated significant perfusion to the cardiac mass (see Supplementary material online, *Video S9*). Delayed myocardial enhancement (MDE) sequences demonstrated diffuse MDE in the cardiac mass suggesting that the cardiac mass was not thrombus, lipoma, or cyst (*Figure 3E*).

CT imaging of the chest, abdomen, and pelvis with intravenous contrast did not demonstrate any evidence of a separate primary malignancy that could have metastasized to the heart (see Supplementary material online, *Appendix*, Supplementary material online, *Figure S1A*–*E*). The cardiac mass did not demonstrate any calcification on CT imaging. The mass had a radiodensity of 74 Hounsfield Units (HU) which was not consistent with fat or fluid as in lipoma or cyst, respectively (see Supplementary material online, *Appendix*, Supplementary material online, *Figure S1B*).

Cardiac magnetic resonance findings (*Table 1*) suggested a secondary metastatic tumour, or primary malignant tumour such as angiosarcoma or rhabdomyosarcoma. Primary benign cardiac tumours such as haemangioma or myxoma were deemed less likely. Given the patient’s history of melanoma excision, consideration for metastatic melanoma was raised. As recommended by the oncology team, tissue diagnosis was performed to provide definitive diagnosis. This was particularly important as the proposed treatment for metastatic melanoma (immunotherapy) was different than for sarcomas (vincristine–dactinomycin–cyclophosphamide).

**Table 1:** LV mass tissue characterization on CMR

Tissue characterization sequence	Signal relative to myocardium/sequence appearance
T2-weighted	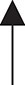
T1-weighted	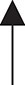
Inversion recovery—fat suppression	No fat suppression
Inversion recovery—thrombus suppression	No suggestion of thrombus
Perfusion	Significant perfusion
MDE	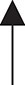

MDE, delayed myocardial enhancement.

**Figure 4:**
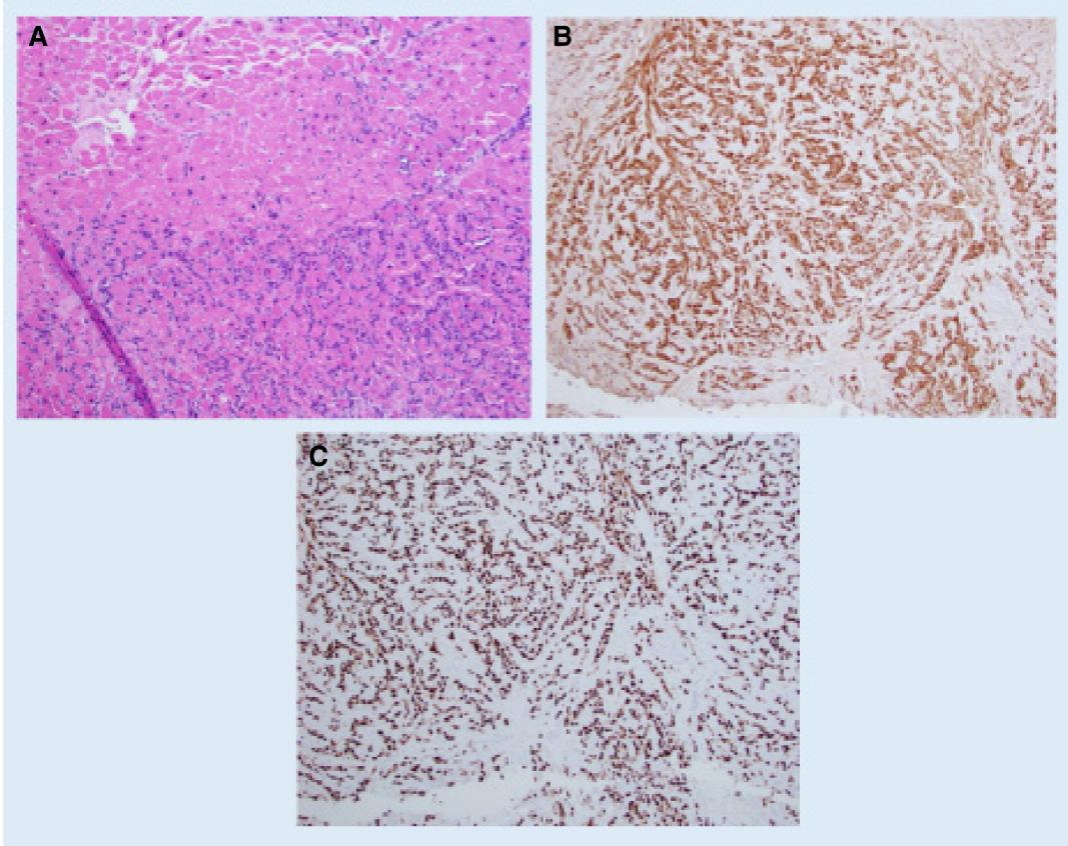
(*A*) H&E section showing malignant cells (dense blue cells) infiltrating the interstitium of the cardiac tissue (pink). (*B* and *C*) Immunostains positive for S100 and SOX 10.

The patient’s hospital course was notable for VT storm refractory to antiarrhythmic therapy (which included amiodarone and lidocaine IV infusions) and ultimately requiring a stellate ganglion block (see Supplementary material online, *Appendix*, Supplementary material online, *Figure S2*). Beta-blocker was not administered due to resting sinus bradycardia. Ventricular tachycardia morphology (relatively narrow with left superior axis and late precordial reverse) was consistent with her presenting ECG (*Figure 1*) with exit site in a more basal posteroseptal wall, correlating with the location of the LV mass. Ablation was not considered due to the thickness and potential friability of the LV mass. After multidisciplinary discussion with interventional cardiology, heart failure, and cardiothoracic surgery teams, a percutaneous endomyocardial biopsy was deemed too high risk for similar reasons. Complete surgical resection was not feasible given the location of the mass in the left ventricle. Therefore, the patient underwent open surgical biopsy and septal myomectomy via open sternotomy and approach through Waterson’s groove with a 2 cm × 1 cm specimen excised. Pathology demonstrated malignant cells infiltrating the cardiac tissue interstitium with features of metastatic melanoma (*Figure 4*). Post-biopsy, the patient required placement of a dual-chamber implantable cardioverter-defibrillator for sinus node dysfunction and sustained VT prior to hospital discharge. The patient was discharged on oral amiodarone.

After diagnosis of metastatic cardiac melanoma, the patient was started on immunotherapy with ipilimumab and nivolumab. With her outside oncologist, she had a whole body 18-fluorodeoxyglucose positron emission tomography (18-FDG-PET) without other sites of avidity apart from the cardiac mass. Cardiac magnetic resonance three months following initiation of immunotherapy demonstrated stable size of the cardiac mass. She reported improvement in dyspnoea and palpitations. Her immunotherapy regimen was adjusted to monthly nivolumab. Following this, she had ICD discharge four times for incessant ventricular tachycardia and underwent outpatient radiofrequency ablation of VT origination from three different foci in the interventricular septum and right ventricular apex. Two months following this, she had repeat PET/CT that demonstrated decreased size of the LV mass. She continued nivolumab maintenance therapy. At her most recent follow-up visits, the patient had repeat PET/CT and CMR demonstrating increased size of the cardiac mass. She was initiated on Opdualag (nivolumab/relatlimab) immunotherapy treatment.

## Discussion

Cardiac tumours can be found incidentally or present with systemic symptoms, haemodynamic instability due to compression of cardiac structures, arrhythmias, or embolic phenomena.^1^ Multimodality cardiac imaging is essential in the evaluation of cardiac tumours, and tissue characterization—particularly with CMR—can help identify malignant cardiac tumours.^2,3^ Few cardiac masses appear hyperintense relative to myocardium on T1W images. Hyperintense T1 signals can be seen with angiosarcomas, metastatic melanomas, lipomas, or recent thrombi.^1^ The cardiac mass had significant perfusion and MDE which is not typically seen with lipoma or thrombus. Secondary cardiac tumours from metastasis apart from melanoma usually appears hypointense on T1W imaging.^1^ Cardiac melanoma is theoretically hyperintense on T1W and hypointense on T2W imaging due to the paramagnetic effects of melanin which cause T1 shortening.^1^ However, in most clinical scenarios, cardiac melanoma appears hyperintense on both T1W and T2W images.^4^

Given the higher prevalence, lung, breast, and oesophageal cancers are more common primary malignancies with cardiac metastases. However, metastatic melanoma has one of the highest propensities for cardiac involvement.^1,4,5,6^ Cardiac melanoma often presents in the left ventricle but can involve any cardiac structure.^6^ Histologic characterization of suspected malignant cardiac masses is necessary. This can be accomplished least invasively if there are sites of extracardiac involvement amenable to sampling, or if there is associated pericardial fluid that can be sampled by pericardiocentesis. Surgical excision of cardiac masses may be indicated for masses with solitary or localized involvement, significant haemodynamic compromise, or potential for embolization.^1,7^ Surgical excision of malignant secondary cardiac tumours, and specifically, melanoma involving the ventricle, can be an adjunct to medical therapies.^7,8^ Cardiac involvement of melanomas typically occurs in end-stage disease—most of such cases are only identified at autopsy. Therefore, recommendations regarding surgical management for our patient were limited to case reports.^4–6^ 18-Fluorodeoxyglucose positron emission tomography can be used to identify extracardiac sites of metastatic melanoma and monitor response to immunotherapy.^9^ Our patient did not experience any recognized side effects of immunotherapy, however, it is important to monitor for potential side effects such as myopericarditis, cardiac dysfunction, arrhythmias, or myocardial infarction.^10^

Metastatic melanoma should be on the differential for cardiac masses. Any patient presenting with a cardiac mass should be asked about history of skin malignancy. Multimodality imaging is crucial to diagnosis, staging, haemodynamic assessment, interventional and surgical planning, and assessment of response to therapy. Multidisciplinary team involvement is essential in the evaluation and management of isolated LV masses.

## Supplementary Material

ytae505_Supplementary_Data

## Data Availability

The data underlying this article are available in the article and in its online Supplementary material.

## Lead author biography

Hussain Khalid, MD, is a clinical assistant professor in the division of cardiovascular medicine at the University of Florida. He earned his medical degree from the University of Florida College of Medicine where he graduated with Honors in Research, and was awarded the 2016 ‘C. Richard Conti Award for Academic Excellence in the field of Cardiovascular Medicine’. He completed his residency training in Internal Medicine at Case Western Reserve University where he was awarded the Resident Award for Research in 2018. He completed Cardiology Fellowship training and Advanced Cardiac Imaging Fellowship at the University of Florida where he gained additional expertise in echocardiography, cardiac CT, and cardiac MRI.

## Supplementary material

**Consent:** Patient consent was obtained for the publication of this manuscript in compliance with COPE guidelines.

**Funding:** None declared.
