# Supplementary material for: Cardiac metastatic melanoma presenting with ventricular tachycardia: a multimodality imaging evaluation case report
Source: Eur Heart J Case Rep. 2024 Sep 14;8(10):ytae505. doi: 10.1093/ehjcr/ytae505 (PMC11462447; doi:10.1093/ehjcr/ytae505)
Supplement: ytae505_Supplementary_Data [file ytae505_supplementary_data.zip › Supplemental Appendix 01-07-2024.docx]

**Supplemental Appendix**

**Figure 1: CT Chest, Abdomen, and Pelvis with Contrast**


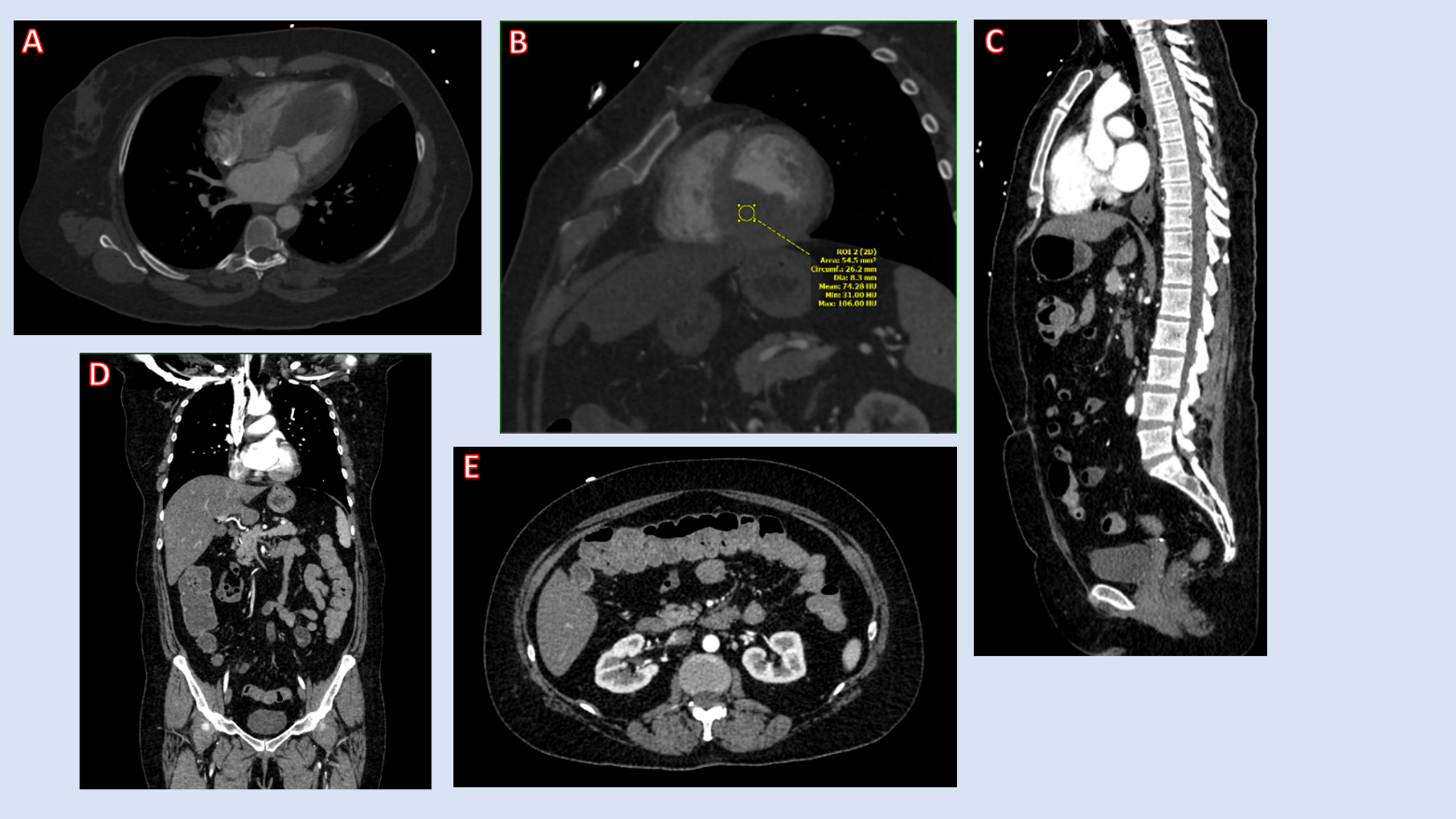


**Figure 1.** A, B, C, D, E: CT axial, coronal, and sagittal images demonstrating cardiac mass and no other extracardiac mass. A,B: The cardiac mass had no calcification. Radiodensity of the cardiac mass was 74 HU.

**Figure 2: Telemetry Strip Demonstrating Monomorphic Ventricular Tachycardia**


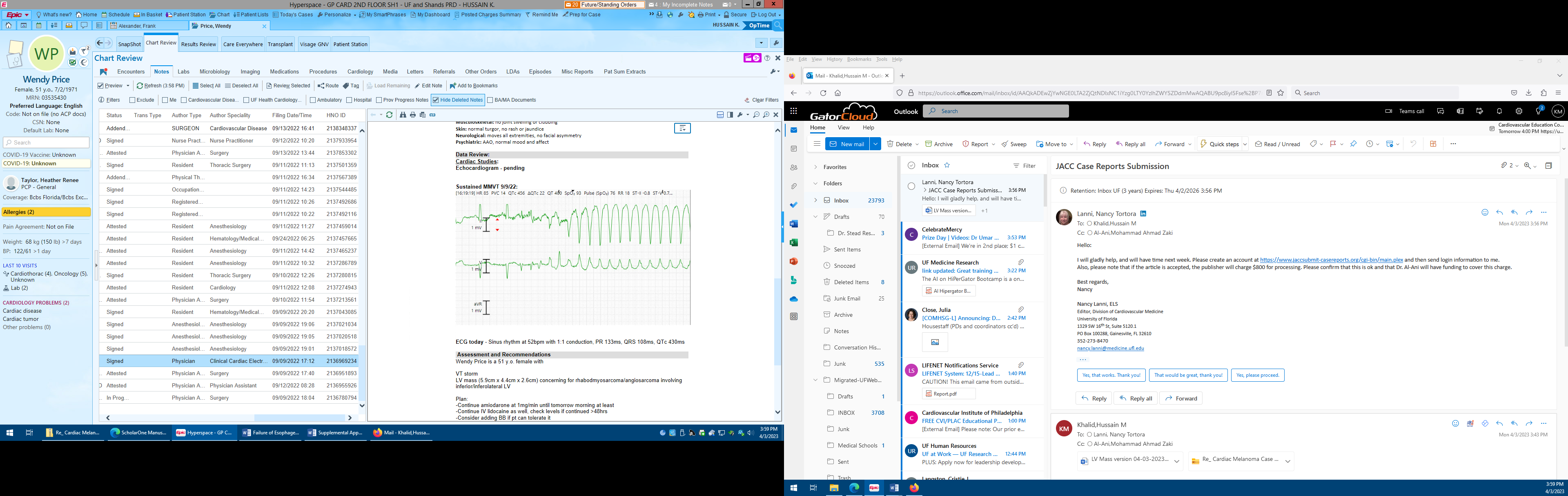


**Figure 2: [Figure 2.** Telemetry strip demonstrating onset of sustained monomorphic ventricular tachycardia.]
