# Supplementary material for: Cardiac metastatic melanoma presenting with ventricular tachycardia: a multimodality imaging evaluation case report
Source: Eur Heart J Case Rep. 2024 Sep 14;8(10):ytae505. doi: 10.1093/ehjcr/ytae505 (PMC11462447; doi:10.1093/ehjcr/ytae505)
Supplement: ytae505_Supplementary_Data [file ytae505_supplementary_data.zip › Video Descriptions01-07-2024.docx]

**Video Descriptions**

**Video 1:**

IV Definity® contrast-enhanced A4C view demonstrating an LV mass involving the mid-to-basal inferoseptum with mild contrast uptake.

**Video 2:**

Color Doppler in the A2C view demonstrating mild mitral regurgitation.

**Video 3:**

IV Definity® contrast-enhanced A2C view demonstrating an LV mass involving the mid-to-basal inferior wall with mild contrast uptake.

**Video 4:**

IV Definity® contrast-enhanced SAX view demonstrating an LV mass involving the mid-to-basal inferoseptum and inferior walls with mild contrast uptake.

**Video 5:**

IV Definity® contrast-enhanced modified PLAX view demonstrating an LV mass involving the mid-to-basal inferoseptum with mild contrast uptake.

**Video 6:**

4- chamber FIESTA sequences demonstrating normal biventricular systolic function and an LV mass. TE 1.9, TR 4.2.

**Video 7:**

2- chamber FIESTA sequences demonstrating LV mass and mildly restricted posterior leaflet motion with associated mild mitral regurgitation. TE 1.9, TR 4.2.

**Video 8:**

3- chamber FIESTA sequences demonstrating LV mass. TE 1.9, TR 4.2.

**Video 9:**

Short-axis FGRE Time Course demonstrating significant perfusion to the cardiac mass. TE 1.4, TR 3.0.
